# PDDA-Montmorillonite Composites Loaded with Ru Nanoparticles: Synthesis, Characterization, and Catalytic Properties in Hydrogenation of 2-Butanone

**DOI:** 10.3390/polym10080865

**Published:** 2018-08-04

**Authors:** Ewa M. Serwicka, Małgorzata Zimowska, Dorota Duraczyńska, Bogna D. Napruszewska, Małgorzata Nattich-Rak, Grzegorz Mordarski, Lidia Lityńska-Dobrzyńska, Helena Palkova

**Affiliations:** 1 Jerzy Haber Institute of Catalysis and Surface Chemistry, Polish Academy of Sciences, Niezapominajek 8, 30-239 Krakow, Poland; nczimows@cyf-kr.edu.pl (M.Z.); ncduracz@cyf-kr.edu.pl (D.D.); ncnaprus@cyf-kr.edu.pl (B.D.N.); ncnattic@cyf-kr.edu.pl (M.N.-R.); ncmordar@cyf-kr.edu.pl (G.M.); 2 Institute of Metallurgy and Materials Science, Polish Academy of Sciences, Reymonta 25, 30-059 Krakow, Poland; l.litynska@imim.pl; 3 Institute of Inorganic Chemistry, Slovak Academy of Sciences, Dúbravská cesta 9, SK-845 36 Bratislava, Slovakia; helena.palkova@savba.sk

**Keywords:** polymer-clay composite, montmorillonite, polydiallyldimethylammonium, Ru nanoparticles, Ru catalyst, 2-butanone hydrogenation

## Abstract

The effect of synthesis parameters on the physicochemical properties of clay/ polydiallyldimethylammonium (PDDA)/Ru composites and their applicability in hydrogenation of 2-butanone under very mild conditions (room temperature, atmospheric pressure, and aqueous solution) was studied. Three synthetic procedures were employed, differing in the order of addition of components and the stage at which metallic Ru species were generated. The materials were characterized with XRD (X-ray diffraction), XRF (X-ray fluorescence), EDS (energy-dispersive spectroscopy), AFM (atomic force microscopy), TEM/HRTEM (transmission electron microscopy/high resolution transmission electron microscopy), and TG/DSC (thermal gravimetry/differential scanning microscopy techniques. The study revealed that the method of composite preparation affects its structural and thermal properties, and controls the distribution and size of Ru particles. All catalysts are active in hydrogenation of 2-butanone. For best catalytic performance (100% conversion within 30 min) both the size of Ru particles and the load of polymer had to be optimized. Superior catalytic properties were obtained over the composite with intermediate crystal size and intermediate PDDA load, prepared by generation of metallic Ru species in the polymer solution prior to intercalation. This method offers an easy way of controlling the crystal size by modification of Ru/PDDA ratio.

## 1. Introduction

The use of nanoparticles in the design of catalysts based on noble metals is particularly attractive, because the large surface to volume ratio characteristic of very fine metal species enables efficient utilization of costly active phase [1]. In addition, tailoring of metal nanoparticle size offers means for control of catalytic activity [2,3]. Unfortunately, naked nanoparticles are prone to agglomeration, which hinders their effective use [3,4]. This obstacle may be overcome by deposition of metal nanoparticles on an appropriate support, and/or by stabilizing their dispersion with use of various capping agents [3,4,5,6,7]. To this end, the use of polymers containing functional groups that provide attractive interaction with nanoparticles is of particular interest [8]. In such systems the polymer component affords, on one hand, the stabilizing steric bulk framework, and on the other, offers many binding sites capable of simultaneous interaction with nanoparticles. The latter makes nanoparticle immobilization very efficient, even if the interaction with an individual functional group is only weak. The use of polymers as stabilizing agents is of particular advantage in the design of advanced nanomaterials based on clay-related layered silicates, because the latter readily form hybrid structures with polymeric components [9,10]. Clay–polymer composites are well recognized for their industrial applications, ranging from heat resistant automotive components to packaging, coating and adhesive materials, aerospace, optical, electronic, and medical devices. Clay minerals, which belong to the family of layered silicates, are especially attractive for the design of composite materials due to their large abundance in nature and unique structural features. The silicate layers, which are the fundamental units of clay minerals, may be easily used as ready-made building blocks in advanced nanocomposite design [11,12,13,14]. In particular, synthesis of ternary systems involving clay, polymer and various metal nanoparticles (e.g., Ag, Au, Rh, Pd, and Fe), as potential catalysts have been reported [15,16,17,18,19,20].

In the present work we focused our attention on the use of polydiallyldimethylammonium (PDDA) chloride as a polymer stabilizing the catalytically active ruthenium nanoparticles embedded in montmorillonite clay. Montmorillonite is a naturally occurring phyllosilicate mineral, whose lattice is composed of stacked layers built of one octahedral Al-based sheet sandwiched between two Si-based tetrahedral sheets. Isomorphic substitution within the octahedral layer (mainly of Al^3+^ by Mg^2+^) generates a negative charge, which is compensated by the presence of hydrated cations in the interlayer [21]. These cations undergo facile exchange, which is the basis for rich intercalation chemistry of montmorillonite. PDDA is a cationic polyelectrolyte, used as a coagulant in water purification, as an additive controlling disturbing substances in the paper production, or as a model compound in different areas of polyelectrolyte research [22]. It is particularly suitable for formation of composites with montmorillonite, because the PDDA polycations readily enter the mineral structure via exchange with the charge compensating cations [15,16]. The binding of the composite components occurs through electrostatic interactions between the negatively charged sites of clay mineral layers and positively charged groups of the polymer. Ruthenium nanoparticles, the third component of the investigated composites, are known for their catalytic properties, especially in hydrogenation reactions [23]. The aim of this study was to investigate the effect of synthesis parameters on the physicochemical properties of clay/PDDA/Ru composites and their applicability in hydrogenation of 2-butanone under very mild conditions (room temperature, atmospheric pressure, and aqueous solution). Hydrogenation of 2-butanone is a very useful test reaction, because under the adopted conditions it is 100% selective to 2-butanol, and enables straightforward comparison of catalysts activities without the necessity of addressing the selectivity issue [24].

## 2. Materials and Methods 

### 2.1. Materials

The starting montmorillonite used for the preparation of composites, denoted Mt, was the sodium form of the less than 2 μm particle size fraction separated by sedimentation from Jelšový Potok (Envigeo Inc., Slovakia, Czech Republic) bentonite, with cation exchange capacity (CEC) of 85 meq/100 g. 20.0 wt % aqueous solution of low molecular weight polydiallyldimethylammonium (PDDA) chloride (Sigma-Aldrich, Poznań, Poland), average *M_W_* = 100,000–200,000, was used after dilution to 0.05 wt % concentration. RuCl_3_·*x*H_2_O (Sigma-Aldrich) was used as ruthenium source.

**Figure 1:**
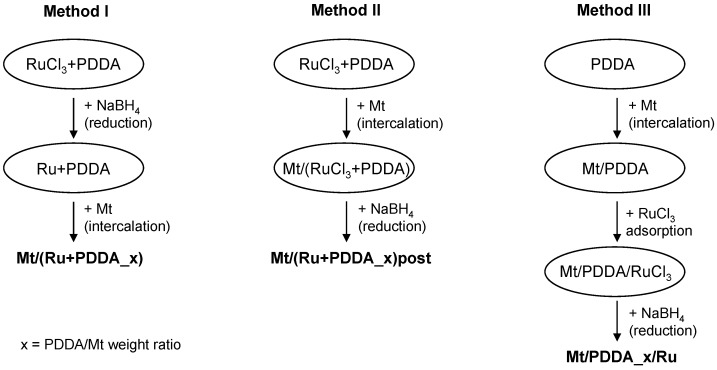
Schematic illustration of catalysts synthesis methods.

The first step in the synthesis of Mt/PDDA/Ru composites involved preparation of Mt suspension in distilled water (0.5 g clay/100 mL water). The suspension was stirred for 24 h at room temperature to enhance Mt exfoliation. Next, 0.2 wt % RuCl_3_ aqueous solution in the amount corresponding to 2 wt % of Ru with respect to Mt component, was mixed with different amounts of 0.05 wt % PDDA aqueous solution for 1 h and reduced with excess NaBH_4_ methanolic solution (molar ratio NaBH_4_/Ru = 100). The mixture was added drop-wise to the Mt suspension at room temperature and left under stirring for 24 h. Then the product was retrieved and washed free of Cl^-^ by centrifugation till negative reaction of the supernatant with AgNO_3_, and stored as aqueous gel. This procedure is referred to as method I. Samples prepared this way, are denoted Mt/(Ru + PDDA_*x*), where *x* is the PDDA/Mt weight ratio, equal 0.01, 0.025, 0.05, 0.1, or 0.25. Theoretically, the complete cation exchange requires the PDDA/Mt weight ratio of 0.14. For the sake of comparison, additional samples with PDDA/Mt ratio of 0.05 and 0.25 were obtained, by changing the sequence of synthetic steps. Thus, in method II, the preparative route followed the path of method I, except that the reduction with NaBH_4_ was carried out in a post-synthesis manner, in the final stage of the composite manufacturing. The resulting two samples are referred to as Mt/(Ru + PDDA_*x*)post. In method III the Mt component was first intercalated with polycations by mixing with the PDDA solution and stirred for 24 h. Subsequently, the resulting Mt/PDDA_*x* complex was mixed with RuCl_3_·*x*H_2_O aqueous solution (2 wt % of Ru with respect to Mt) for 24 h and the whole mixture subjected to reduction with NaBH_4_. The samples obtained this way are referred to as Mt/PDDA_*x*/Ru. The main steps of each procedure are illustrated in Figure 1.

### 2.2. Methods

X-ray diffraction (XRD) patterns were recorded using X’Pert PRO MPD diffractometer (PANalytical, Almelo, the Netherlands) with CuKα radiation (40 kV, 30 mA) selected by a nickel monochromator in the diffraction beam, with a step size 0.05°. The samples were prepared as oriented thin films by drying a suspension of clay composite on a glass slide.

X-ray fluorescence (XRF) analysis, used for the determination of Ru content, was carried out with Skyray EDX 3600 H spectrometer (Skyray, Braintree, MA, USA), equipped with X-ray source (40 kV, 220 μA). The Na/Si ratio in the investigated samples was determined with aid of a JSM-7500F Field Emission scanning electron microscope (JEOL, Akishima, Japan) equipped with an energy-dispersive (EDS) analyzer (Link ISIS, Oxford Instruments, London, UK).

Atomic force microscopy (AFM) measurements of Ru nanoparticles adsorption on mica surface were carried out using the NT-MDT IX71 device with the SMENA scanning head (NT-MDT, Russia). The measurements were performed in semi-contact mode using silicon probes. Ru suspension in PDDA aqueous solution was allowed to adsorb on mica surface for 60 min and then the substrate was removed and rinsed for 30 s with distilled water.

Transmission and high resolution transmission electron microscopic studies (TEM and HRTEM, respectively) were performed with FEI Tecnai G2 (FEI, Eindhoven, the Netherlands) transmission electron microscope. The dominant size of Ru particles in the sample was estimated from the assessment of at least 250 particles in selected areas of enlarged TEM micrographs. 

Thermal analysis was carried out with a DSC/TG Netzsch STA 409 PC LUXX apparatus (Netzch, Germany), in the temperature range 30–1000 °C, at a heating rate of 10 °C /min and in the flow of air (40 mL/min). Theoretical weight losses were calculated under the assumption that the mass loss in the 200–1000 °C range is due to the elimination of the organic component (PDDA) and to the dehydroxylation of Mt layers. The quantitative contribution of the latter was calculated from the TG curve of Mt sample. PDDA is a polymer of 100% cationicity, therefore for the calculation of the initial mass of PDDA-exchanged samples it was assumed that the exchange involves replacement of one Na^+^ with one PDDA^+^ monomer.

2-Butanone hydrogenation experiments were carried out in an agitated batch glass reactor (PARR 5100, Parr Instrument Company, Moline, IL, USA) at constant pressure of hydrogen (1 bar) and room temperature. In a typical hydrogenation test the catalyst, containing 4 × 10^−6^ mole of Ru, was added to the solution of 3 × 10^−3^ mole of 2-butanone in 50 mL water. Prior to the catalytic test the system was purged with nitrogen. Subsequently hydrogen was introduced and the reagents vigorously stirred (800 rpm) for 0.5 h. A gas chromatograph (Clarus 500, Perkin Elmer, Boston, MA, USA) equipped with CP-Chirasil-Dex CB column (Varian Inc., Walnut Creek, CA, USA) was used for analyzing the composition of reaction mixture. Catalytic results were not affected by an increase of the agitation speed, which pointed to the absence of diffusion limitations.

## 3. Results and Discussion

### 3.1. Physicochemical Characterization

**Figure 2:**
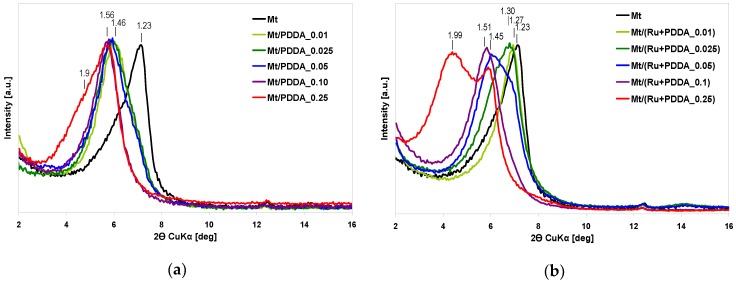
(**a**) Effect of polydiallyldimethylammonium (PDDA) loading on the XRD pattern of parent montmorillonite; (**b**) Effect of Ru insertion by method I on XRD pattern of montmorillonite (Mt) intercalated with PDDA cations (PDDA loadings as in Figure 2a).

Prior to studying the Ru-containing composites, the effect of PDDA intercalation on the XRD pattern of the parent Mt was examined (Figure 2a).

The basal spacings of PDDA-intercalated samples, reported in Table 1, show a shift from *d*_001_ = 1.23 nm, characteristic of the sodium form of montmorillonite, to 1.46–1.56 nm, typical of PDDA-clay complex in which a single layer of polymer chains, adhering to the basal planes of montmorillonite, is formed [25,26,27]. The effect is associated with the 100% cationicity of PDDA, which causes strong attraction with clay layers and favors extended polymer conformation, with few loops and trains [26]. Noteworthy, in samples with the lowest PDDA loadings, the basal reflection displays an asymmetry in the direction of higher *2Θ* values, towards the basal reflection of the parent clay mineral, indicating the presence of not completely exchanged interlayers. Indeed, the chemical analysis shows that these samples retain a certain amount of sodium. This is not surprising, since in most samples the amount of PDDA used for intercalation was less than the CEC (Table 1). However, the data in Table 1 show that the Na content, especially in samples with less PDDA, is much lower than that expected to be left in the materials after complete exchange with PDDA cations (the amount of PDDA used in the synthesis corresponded to ca. 7%, 18%, and 36% of CEC for Mt/(Ru + PDDA_0.01), Mt/(Ru + PDDA_0.025), and Mt/(Ru + PDDA_0.05), respectively. Presumably, in view of the acidic pH of PDDACl solution (pH = 2), a part of sodium becomes exchanged with hydronium ions. Under the adopted conditions the interlayer spacing of H-form of montmorillonite is ca. 1.5 nm, therefore we propose that the basal reflection corresponding to this value is, especially in samples with low PDDA loadings, partly due to the presence of H_3_O^+^ in the Mt interlayer. In the Mt/PDDA_0.25 sample, loaded with the highest PDDA content employed in this work a shoulder at lower *2Θ* appears, corresponding to ca. 1.9 nm interlayer distance, suggesting that a part of intercalated polymer forms a double layer [26,27].

In the case of method I, in which the reduced Ru species were formed in PDDA prior to intercalation, dispersion of metal particles in the polymer solution has been examined with aid of atomic force microscopy. 

**Figure 3:**
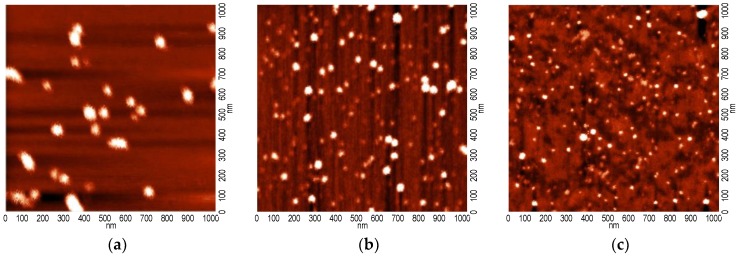
AFM topography images of Ru suspensions in PDDA solutions used for intercalation of (**a**) Mt/(Ru + PDDA_0.01); (**b**) Mt/(Ru + PDDA_0.05); and (**c**) Mt/(Ru + PDDA_0.25) samples.

Figure 3 shows the representative AFM images recorded for three PDDA loadings: the minimum, the intermediate and the maximum one (*x* = 0.01, 0.05, and 0.25). It may be seen that with the increase of the relative content of PDDA, Ru nanoparticles become better dispersed. The size of the smallest particles visible in the AFM images is about 20 nm. Such species dominate in the intercalating agent used for Mt/(Ru + PDDA_0.25) preparation, but upon lowering of the relative PDDA content the particles tend to form larger agglomerates, of ca. 60–80 nm dimension, and such clusters prevail in the suspension used for Mt/(Ru + PDDA_0.01) synthesis. The complex nature of AFM-visible particles is confirmed by the TEM study, described further.

The influence of Ru addition by method I on the structural features of PDDA/clay intercalates is illustrated in Figure 2b. It may be seen that generation of Ru species in the polymer solution prior to intercalation affects the manner of polymer insertion and causes differentiation of the observed basal spacings. In particular, in the case of samples with lower PDDA loadings, intercalation appears to be hindered, as indicated by d_001_ values lower than in the corresponding Ru-free organomontmorillonites (Figure 2a, Table 1). In the case of samples Mt/(RU + PDDA_0.05) and Mt/(Ru + PDDA_0.25) the products of intercalation show structural inhomogeneity. In the first case a shoulder at higher *2Θ* values indicates that there are polymer-poor regions in the final composite, in the second, next to the peak corresponding to d_001_ value of 1.50 nm, a strong reflection associated with d_001_ = 1.99 nm shows that in a substantial part of Mt interlayers the intercalated polymer forms a double layer. In order to explain inhibition of PDDA intercalation in Ru catalysts with low PDDA content it should be recalled that ruthenium is generated within PDDA solution by treatment with large excess of NaBH_4_, which introduces into the solution a load of sodium cations, corresponding to ca. 5× CEC of montmorillonite. Thus, competition with Na^+^ prevents efficient intercalation of PDDA cations between the clay layers, the more, the lower is the polycation concentration. At higher PDDA contents (x ≥ 0.05), the well-known affinity of cationic polymer to clay matrix ensures that intercalation does occur, despite the fact that sodium concentration in the solution still exceeds that of PDDA polycations. Remarkably, in the sample with the highest nominal PDDA content, a substantial part of polymer assumes a bilayer packing (d_001_ = 1.99 nm) much more pronounced than in the Ru-free sample.

**Figure 4:**
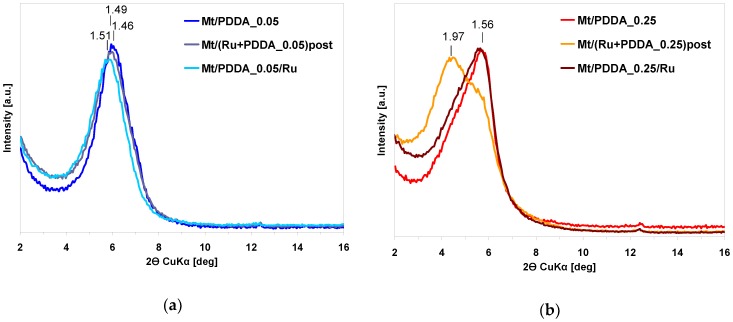
(**a**) Effect of Ru insertion by method II and method III on XRD pattern of Mt intercalated with PDDA cations (*x* = 0.05); (**b**) effect of Ru insertion by method II and method III on XRD pattern of Mt intercalated with PDDA cations (*x* = 0.25).

The effect of Ru insertion by method II and III on XRD pattern of organomontmorillonite was studied for two levels of PDDA loadings, *x* = 0.05, and *x* = 0.25, and is shown in Figure 4a,b, respectively. In the former case, the XRD diagrams of Ru-containing composites do not differ in any significant manner from that of Mt/PDDA_0.05 sample, suggesting a similar manner of polymer packing in all samples. For *x* = 0.25, addition of ruthenium by method II causes, similarly as in the case of method I, evolution, next to the peak corresponding to *d*_001_ = 1.56 nm, of a reflection associated with *d*_001_ = 1.97 nm, indicative of clay intercalated by a double layer of polymer. Thus, for the XRD-detectable structural order it is irrelevant whether Ru added to the polymer solution is reduced prior to (method I) or after (method II) intercalation. Application of method III to Ru insertion does not affect the XRD pattern of the parent Mt/PDDA samples in any meaningful way.

In none of the Ru-containing samples can any reflections of metallic Ru be observed, which suggests that either the absolute content of Ru is too low to be detected by XRD, and/or that nanocrystalline character of Ru particles results in broadening of the diffraction peaks beyond possible detection.

**Figure 5:**
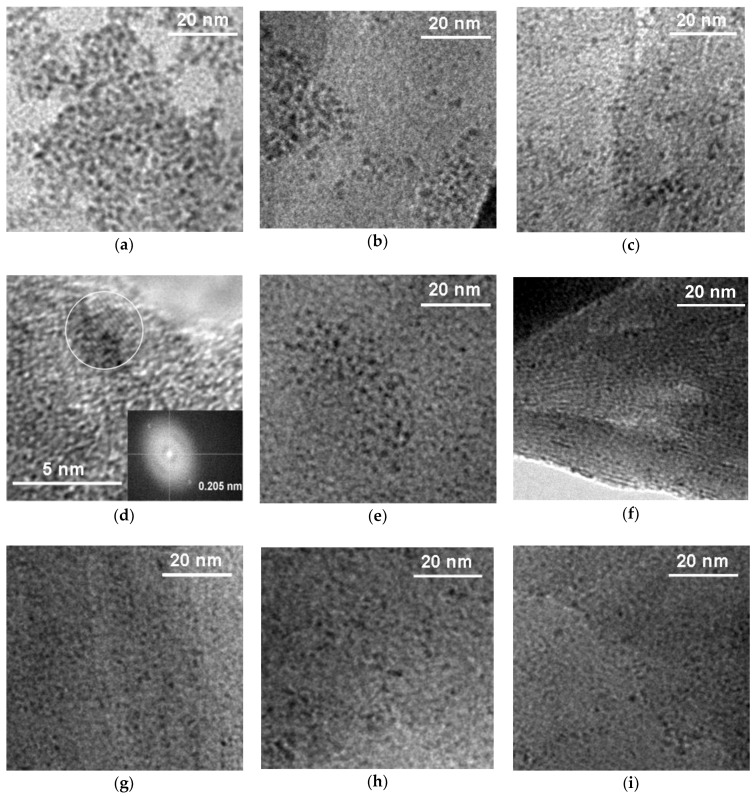
TEM images of Ru particles in: (**a**) Mt/(Ru + PDDA_0.01); (**b**) Mt/(Ru + PDDA_0.05); (**c**) Mt/(Ru + PDDA_0.25); (**d**) HRTEM image of Ru crystallite in Mt/(Ru + PDDA_0.05) with corresponding FTT; (**e**,**f**) Mt/(Ru + PDDA_0.05)post; (**g**) Mt/PDDA_0.05/Ru; (**h**) Mt/(Ru + PDDA_0.25)post; (**i**) Mt/PDDA_0.25/Ru.

TEM analysis of samples prepared by method I (Figure 5a–d) reveals that particles observed by AFM are, in fact, composed of much finer crystallites, of sizes in the range 2–4 nm, the larger ones being preferentially formed in samples with low relative polymer content, the finer in materials rich in polymer component (Figure 5a–c). The effect is understandable in terms of the stronger dilution of RuCl_3_ precursor in the larger volumes of aqueous solution of PDDA. Moreover, in accordance with AFM data, upon decrease of the polymer content the Ru crystallites tend to form larger agglomerates. Figure 5d shows a HRTEM image of Ru crystallite with noticeable lattice fringes, representative for particles found in Mt/(Ru + PDDA_0.05) sample. The interplanar distance of 0.205 nm, derived from the fast Fourier transform (FFT), corresponds to (10–11) plane of hexagonal Ru lattice (ref. code 04-007-8640). Comparison of Figure 5e,g, which show Ru particles in samples Mt/(Ru + PDDA_0.05)post and Mt/PDDA_0.05/Ru, with Figure 5b, showing the Mt/(Ru + PDDA_0.05) material (all samples with the same nominal weight ratio of all components) enables assessment of the influence of various methods of Ru insertion on the form of generated ruthenium species. Thus, for PDDA/clay ratio of 0.05, the preparation of the catalysts by mixing Ru source with the polymer solution followed by reduction prior to intercalation (method I) yields relatively largest Ru particles (dominating size ca. 3 nm), the procedure with post-synthesis reduction of clay intercalated with polymer containing Ru precursor (method II) produces somewhat smaller crystallites (dominating size 2–2.5 nm), while adsorption of Ru on previously intercalated montmorillonite (method III) results in smallest, most dispersed species (dominating size 1–1.5 nm). Moreover, in sample prepared by method II, which enables co-intercalation of Ru^3+^ into the interlayer space, formation of very small Ru particles (<1 nm) in the interlayer space of clay is also observed (Figure 5f). In the case of the maximum PDDA/clay weight ratio of 0.25, comparison of appropriate TEM images shows that all methods of Ru incorporation result in the formation of very small nanoparticles, of ca. 1–2 nm (Figure 5c,h,i).

**Figure 6:**
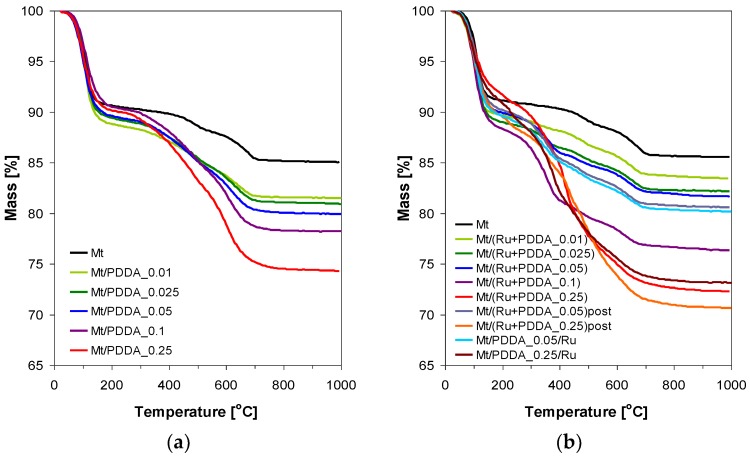
TG profiles of: (**a**) PDDA-intercalated montmorillonites; (**b**) Ru-loaded PDDA-intercalated montmorillonites. TG of parent montmorilllonite is provided for comparison.

**Figure 7:**
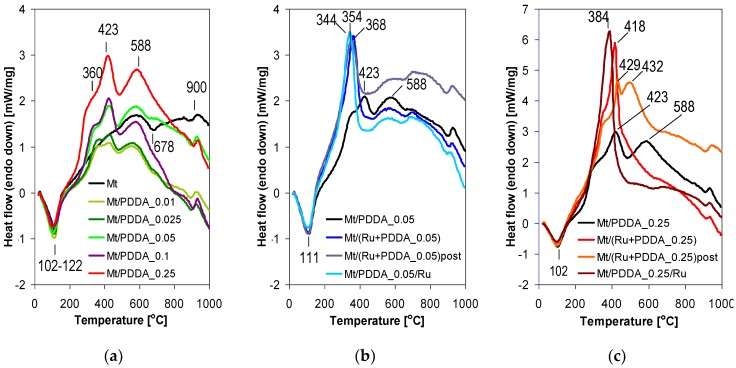
DSC profiles of: (**a**) PDDA-intercalated montmorillonites; (**b**) composites with PDDA/clay weight ratio of 0.05; (**c**) composites with PDDA/clay weight ratio of 0.25.

Investigated samples have been subjected to thermal analysis, in order to get insight into the amount of intercalated polymer (Figure 6 and Figure 7, Table 1). Assignment of the TG/DSC effects follows the established views on the thermal properties of montmorillonite and its organic derivatives [28,29,30]. 

The weight loss in the TG curves of PDDA–montmorillonite complexes occurs in two major temperature ranges, below and above ca. 200 °C (Figure 6a). In the parent montmorillonite the weight loss below 200 °C is associated with the departure of interlayer water coordinating exchangeable Na^+^ ions, with a minor contribution from physisorbed water held in interparticle pores. The weight loss in the range 200–700 °C is due to dehydroxylation of montmorillonite layers reflected in the departure of water. It should be noted that the phenomena responsible for both steps overlap to some extent and there is no clear-cut border between them [31]. Thermal degradation/combustion of the organic component in PDDA-containing samples occurs above 200 °C and overlaps with the dehydroxylation of clay mineral layers. Table 1 provides the values of the observed weight losses in the temperature range 200–1000 °C for all samples whose TG profiles are presented in Figure 6a,b. The theoretical weight losses, estimated under the assumption that all introduced polymer has been incorporated into the sample, are given in brackets. For all samples, except of those with the highest employed PDDA/Mt ratio of 0.25, the experimental values remain within the ±15% margin from the theoretical ones, which, bearing in mind that the end of the clay dehydration step may extend above the arbitrary limit of 200 °C, indicates that practically all PDDA enters the montmorillonite structure (Table 1). This is the case even for the Mt/PDDA_0.01 sample, with the lowest PDDA loading, corresponding to ca. 7% of the clay CEC, and demonstrates the high affinity of the polycation towards the clay matrix. In the case of samples with PDDA/Mt ratio of 0.25, the amount of added PDDA corresponds to ca. 180% of CEC, therefore Table 1 provides also information of the theoretical mass loss expected if the PDDA content in the composite is less than the added total and corresponds to the maximum CEC-allowable value. It may be seen that for Mt/PDDA_0.25 and Mt/PDDA_0.25/Ru samples, in which Ru-free PDDA solution was used for intercalation, the observed weight loss indicates that the samples retain less than the introduced amount of PDDA, close to the CEC dependent value, the excess being apparently removed upon washing. In contrast, in Mt/(Ru + PDDA_0.25) and Mt/(Ru + PDDA_0.25)post samples, in which intercalated PDDA solution contained either Ru particles or RuCl_3_, the amount of incorporated PDDA is higher, suggesting that in the presence of Ru species more PDDA can be loaded into the clay matrix. In both materials the stage of final washing of the sample comes after the formation of Ru nanoparticles. Possibly, the polymer interacting with Ru nanoparticles is more rigid and less prone to removal upon washing than that present in the Ru-free system. The presence of larger amount of polymer also explains the appearance of a bilayer PDDA packing in these samples, evidenced by XRD (Figure 2b and Figure 4b). 

Valuable information as to the nature of the interaction between the components of the composites, depending on the method of Ru insertion, can be derived from the analysis of DSC profiles (Figure 7). In parent Mt (Figure 7a), the endotherm peaking near 110 °C accompanies the release of interlayer water associated with the compensating cations, the endotherm at 678 °C is due to the dehydroxylation of the montmorillonite layers, and a small S-shaped endo-exotherm centered around 900 °C indicates structural rearrangements accompanying the formation of new phases. With addition of PDDA component, new maxima of exothermic character appear, at 360, 423, and 588 °C (Figure 7a). As expected, their intensity increases with the amount of added polymer. The first two are attributed to the decomposition/combustion of PDDA, the third is attributed to the combustion of the carbonaceous deposit formed during incomplete oxidation of the organic matter at the previous stage of polymer destruction. 

The effect of various manners of Ru addition to samples with the PDDA/clay ratio of 0.05 is shown in Figure 7b. The overall appearance of the DSC profiles for Ru-containing samples is similar, irrespective of the preparation method. In all cases it is obvious that in the presence of Ru, the combustion of the PDDA component is shifted to lower temperature, as manifested by the strong exotherm around 350 °C, accompanied by the disappearance/suppression of 423 and 588 cm^-1^ maxima in Ru-free matrix. This effect may be attributed to the catalytic action of Ru nanoparticles, known to facilitate total oxidation of organic matter [32]. Moreover, despite the general similarity of DSC curves for Ru-containing samples with PDDA/clay ratio of 0.05 prepared by different methods, there is a difference between the positions of maxima relevant to PDDA combustion. The ease of PDDA component combustion follows the order: Mt/PDDA_0.05/Ru (344 °C) > Mt/(Ru + PDDA_0.05)post(354 °C) > Mt/(Ru + PDDA_0.05) (369 °C). In view of the TEM data it appears that the smaller the Ru nanoparticles the higher is their efficiency in PDDA component combustion. The effect is understandable in terms of the growing Ru–PDDA interface upon diminution of Ru species.

Thermal properties of samples with the nominal PDDA/clay ratio of 0.25 (Figure 7c) show considerably larger differences. Although the general effect of accelerated combustion of the organic matter, manifested by the downward shift of associated exothermic maxima, is still present, it is not so strong and depends on the manner of Ru insertion. Only in the case of Mt/PDDA_0.25/Ru sample, prepared by method III, and containing considerably less PDDA than the nominal loading, the DSC profile resembles that of the Mt/PDDA_0.05/Ru counterpart. Also here a single exothermic maximum is observed at 384 °C, indicating that efficient combustion, leaving no significant char, occurs below 400 °C. A different situation is found with samples Mt/(Ru + PDDA_0.25) and Mt/(Ru + PDDA_0.25)post, prepared by method I and II. Here, the downward shift is practically limited to the effect related to the combustion of carbonaceous deposit, while the first maximum appears at a similar temperature around 420 °C. The observed phenomena suggest that in the composites most heavily loaded with PDDA, in which the polymer areas in contact with Ru species lose on importance with respect to PDDA fraction not affected by the Ru presence, it is the diffusion of oxygen required for the combustion, that controls the evolution of DSC profiles. Thus, the first DSC effect around 420 °C appears at a similar position as in the Ru-free material, because penetration of polymer-loaded material by oxygen, to reach the Ru-containing areas, is hindered by the large volume of polymer chains. On the other hand, at higher temperature, when most of the PDDA has been burned off and the remnants turned into the carbonaceous deposit, new diffusion paths for air become available, and the effect of Ru species may become visible in the form of lowering of the char combustion temperature, more pronounced in the material with the relatively smaller initial content of polymer, i.e., Mt/(Ru + PDDA_0.25)post.

### 3.2. Catalytic Testing

**Figure 8:**
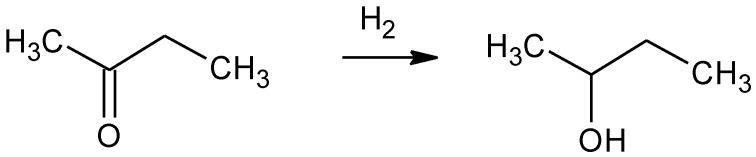
Scheme of 2-butanone hydrogenation.

Among many organic transformations, catalytic hydrogenation of C=O bond is one of the most important synthetic methods enabling the preparation of a great number of useful products [33]. Hydrogenation of 2-butanone yields 2-butanol (Figure 8), a commonly used industrial solvent. 

**Figure 9:**
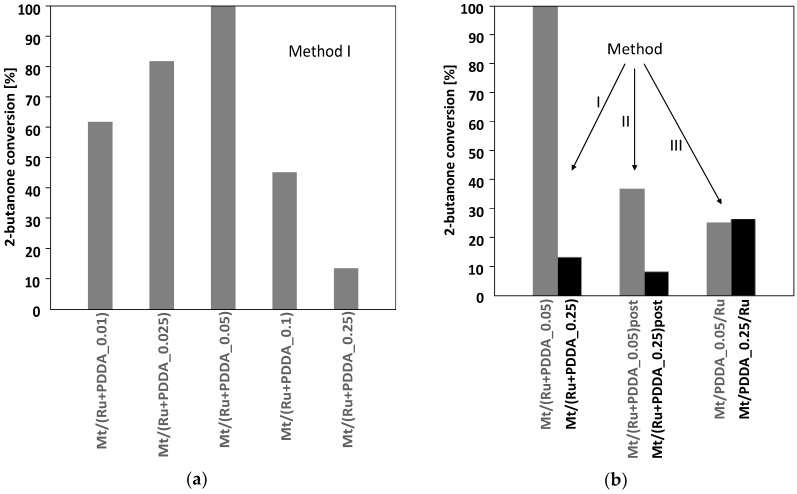
(**a**) Catalytic activity in 2-butanone hydrogenation of Mt/(Ru + PDDA_*x*) composites; (**b**) comparison of catalytic activity of composites prepared by different methods, for polymer/clay weight ratio of 0.05 and 0.25. In all cases selectivity to 2-butanol is 100%.

The results of the catalytic tests of 2-butanone hydrogenation carried out in this work are summarized in Figure 9. Figure 9a shows the dependence of 2-butanone conversion for the series of catalysts obtained by method I, differing in the polymer/clay weight ratio. No conversion of 2-butanone was observed on parent Mt or an organoclay not containing ruthenium. It is apparent that the highest activity (100% conversion) is obtained over the Mt/(Ru + PDDA_0.05) catalyst with an intermediate polymer content. It has been repeatedly demonstrated by theoretical and experimental studies that maximum catalytic activity in various processes catalyzed by Ru nanoparticles can be achieved by optimization of the particle size, so as to obtain the highest population of the active sites favorable for the catalytic transformation [34,35,36]. In the present work, the physicochemical characterization revealed that Ru particle sizes in this series vary in the 2–4 nm range, showing a tendency to become smaller upon increase of the relative polymer content. The maximum activity observed for the catalyst with intermediate PDDA loading, hence intermediate Ru particle dimensions, may be taken as an indication that also in the present study the size of Ru species plays an important role. However, it appears that it is not the only factor influencing the catalytic activity. Figure 9a shows that a particularly rapid fall of activity occurs in materials heavily loaded with PDDA, i.e., Mt/(Ru + PDDA_0.1) and Mt/(Ru + PDDA_0.25). Although in these samples Ru crystallites become smaller, so the fall of activity is in line with the hypothesis of the existence of an optimum Ru crystal size, but it should be remembered that the large volume of polymer present in these materials is likely to hinder the access of reagents to Ru particles. Therefore, the diffusional limitations within the catalyst structure are another probable cause of the observed strong decrease of catalytic activity. 

Comparison of catalytic experiments carried out for composites with the same polymer loading, but prepared with different methods of Ru insertion, sheds additional light on the role of particular factors in determining the catalytic performance of Ru-clay-polymer composites (Figure 9b). Thus, in the case of materials prepared with the PDDA/clay weight ratio of 0.05, which exploits only about 35% of the clay CEC, the order of catalytic activity follows the order of Ru particles size, the most active catalyst being the one obtained by method I, with most particles having ca. 3 nm, followed by samples obtained by methods II and III, with lower particle sizes. In samples with the highest investigated PDDA/clay ratio of 0.25 (loading in excess to CEC), which all contain small Ru particles (≤2 nm), catalytic performance depends strongly on the manner of catalysts preparation. Thus, composites prepared by methods I and II, i.e., Mt/(Ru + PDDA_0.25) and Mt/(Ru + PDDA_0.25)post, perform significantly poorer than their counterparts with PDDA/clay ratio equal 0.05. The effect is attributed to the high polymer content, making it difficult for the reagents to reach Ru particles. In contrast, in the case of sample prepared by method III, Mt/PDDA_0.25/Ru, the catalytic activity is comparable with that observed for the Mt/PDDA_0.05/Ru with much lower PDDA content. Moreover, the sample displays the highest activity among the materials with high PDDA loading. The latter may be due in part to the lower PDDA amount in this sample, causing lesser blocking, and in part to the manner of Ru addition. When Ru is introduced by adsorption onto the already formed Mt/PDDA composite, the Ru particles, although very small, and as such far from being optimally active, are likely to remain at the surface or in the near-to-surface area of the catalyst, thus eliminating diffusional limitations typical of PDDA-rich materials prepared by methods I and II. This explains both the higher activity of the Mt/PDDA_0.25/Ru catalyst in comparison with samples prepared by methods I and II, and the lack of difference in catalytic performance with respect to the Mt/PDDA_0.05/Ru catalyst.

Finally, we would like to underline, that the Ru catalysts based on PDDA/clay composites described in this work are active under exceptionally mild conditions. The vast majority of the reported studies on 2-butanone heterogeneous catalytic hydrogenation have been investigated at elevated hydrogen pressure and/or temperature, while the conditions used in the present work involved normal hydrogen pressure and room temperature [24,37,38,39]. Moreover, all catalysts obtained by method I, with PDDA/clay weight ratio ≤ 0.05, outperform ruthenium catalysts of similar Ru content, supported on mesoporous silica of SBA-15 type and/or conventional high surface area silica, described in our recent work, which, in the same catalytic system, converted 51 and 37 % 2-butanone, respectively [40]. 

## 4. Conclusions

The amount of PDDA retained by montmorillonite is governed by the CEC of clay. When the quantity of added PDDA is below CEC, practically all polycations become trapped by the clay matrix; when it is higher than CEC, most of the excess polymer can be removed at the stage of washing. 

It is postulated that, due to the acidic pH of PDDACl solution, in samples with lower PDDA loading, next to the single layer of polycations, also hydronium ions are intercalated and contribute to the observed basal spacing. 

Generation of Ru species in the polymer solution prior to intercalation (method I) hinders intercalation of polycations into composites with low PDDA/clay ratio, because of the competitive exchange with Na^+^ from the NaBH_4_ solution used for Ru^3+^ reduction. No such effect is observed in composites obtained by procedures in which treatment with NaBH_4_ is carried out after polymer intercalation (methods II and III). 

TEM study reveals that Ru crystallites are quite small, from less than 1 to 4 nm, depending on the preparation method. In preparative routes involving mixing of Ru source with the polycation solution (methods I and II), the size of Ru particles diminishes upon increase of the relative PDDA content. The finest particles are formed upon adsorption of Ru onto clay intercalated with PDDA (method III). 

The presence of Ru changes thermal properties of the polymer–clay composites, as it catalyzes the combustion of the organic component. The effect is more pronounced in materials with lower relative PDDA content and smaller Ru particles, i.e., under conditions providing higher polymer–Ru interface.

The catalysts are active in hydrogenation of 2-butanone under very mild conditions (room temperature, atmospheric pressure of H_2_, and aqueous solution). For best catalytic performance both the size of Ru particles and the load of polymer have to be optimized. Superior catalytic properties are obtained over the composite with intermediate crystal size and intermediate PDDA load, prepared by generation of metallic Ru species in the polymer solution prior to intercalation. This method offers an easy way of controlling the crystal size by modification of Ru/PDDA ratio. 

Results of this study underline the significance of fine details in the preparative procedures aiming at synthesis of ternary clay/polymer/metal nanoparticles composites. The presented findings provide insight into the factors enabling control of the materials properties and are expected to be of use in the design of related systems. 

## Figures and Tables

**Table 1:** Basal spacing, Na/Si atomic ratio, Ru content, mass loss in the 200–1000 °C range, and 2-butanone conversion of studied samples.

Sample	d_001_ (nm)	Na/Si	Ru (wt %)	ΔTG_200-1000_ (wt %)	2-Butanone Conversion (%)
Mt	1.25	0.092	-	5.6	0
Mt/PDDA_0.01	1.46	0.021	-	7.3 (6.3) ^1^	n.d ^2^
Mt/PDDA_0.025	1.49	0.012	-	8.5 (7.4)	n.d.
Mt/PDDA_0.05	1.50	0.009	-	9.7 (9.2)	0
Mt/PDDA_0.1	1.56	0.003	-	12.3 (12.5)	n.d.
Mt/PDDA_0.25	1.53	0.001	-	15.9 (21.3, 15.0 *)	n.d.
Mt/(Ru + PDDA_0.01)	1.27	0.080	1.92	6.2 (6.2)	63
Mt/(Ru + PDDA_0.025)	1.30	0.062	1.85	6.8 (7.3)	82
Mt/(Ru + PDDA_0.05)	1.45 (1.30)	0.044	1.95	8.2 (9.0)	100
Mt/(Ru + PDDA_0.1)	1.51	0.028	1.81	12.0 (12.4)	45
Mt/(Ru + PDDA_0.25)	1.99 (1.50)	0.008	1.67	19.8 (21.3, 14.7 *)	13
Mt/(Ru + PDDA_0.05)post	1.49	0.046	1.74	9.6 (9.0)	36
Mt/(Ru + PDDA_0.25)post	1.97 (1.56)	0.002	1.61	18.9 (21.3, 14.7 *)	7
Mt/PDDA_0.05/Ru	1.51	0.048	1.99	9.4 (9.0)	26
Mt/PDDA_0.25/Ru	1.57	0.001	1.74	16.3 (21.3, 14.7 *)	27

^1^ in brackets weight loss expected if all added polymer entered the composite, ^2^ n.d.—not determined; * weight loss expected if the amount of retained polymer corresponds to the CEC of clay.
